# Development and implementation of a treatment pathway to reduce coronary angiograms - lessons from a failure

**DOI:** 10.1186/s12913-024-10904-5

**Published:** 2024-04-25

**Authors:** Jutta Jung-Henrich, Kathrin Schlößler, Til Uebel, Nino Chikhradze, Anastasia Suslow, Nicole Lindner, Sandra Fahrenkrog, Judith Kraft, Eva Hummers, Horst Christian Vollmar, Ildikó Gágyor, Dirk Heider, Hans-Helmut König, Norbert Donner-Banzhoff

**Affiliations:** 1 Department of General Practice/Family Medicine, Philipps-University Marburg, Karl-Frisch- Straße 4, 35043 Marburg, Germany; 2 Institute of General Practice and Family Medicine (AM RUB), Ruhr University Bochum, Universitätsstraße 150, 44801 Bochum, Germany; 3 Department of General Practice, University Hospital Würzburg, Josef-Schneider-Strasse 2, 97080 Würzburg, Germany; 4 Institute of General Practice and Family Medicine, Charité University Berlin, Charitéplatz 1, 10117 Berlin, Germany; 5 Department of General Practice, Georg-August-Universität Göttingen, Humboldtallee 38, 37073 Göttingen, Germany; 6 Department of Health Economics and Health Services Research, University Medical Center Hamburg-Eppendorf, Hamburg, Germany

**Keywords:** Small area-analysis, Coronary artery disease, Guideline Adherence, Standard of Care [MeSH], Treatment pathway [non-MeSH]

## Abstract

**Background:**

The rates of coronary angiograms (CA) and related procedures (percutaneous intervention [PCI]) are significantly higher in Germany than in other Organisation for Economic Co-ordination and Development (OECD) countries. The current guidelines recommend non-invasive diagnosis of coronary heart disease (CHD); CA should only have a limited role in choosing the appropriate revascularisation procedure. The aim of the present study was to explore whether improvements in guideline adherence can be achieved through the implementation of regional treatment pathways. We chose four regions of Germany with high utilisation of CAs for the study. Here we report the results of the concomitant qualitative study.

**Methods:**

General practitioners and specialist physicians (cardiologists, hospital-based cardiologists, emergency physicians, radiologists and nuclear medicine specialists) caring for patients with suspected CHD were invited to develop regional treatment pathways. Four academic departments provided support for moderation, provision of materials, etc. The study team observed session discussions and took notes. After the development of the treatment pathways, 45 semi-structured interviews were conducted with the participating physicians. Interviews and field notes were transcribed verbatim and underwent qualitative content analysis.

**Results:**

Pathway development received little support among the participants. Although consensus documents were produced, the results were unlikely to improve practice. The participants expressed very little commitment to change. Although this attempt clearly failed in all study regions, our experience provides relevant insights into the process of evidence appraisal and implementation. A lack of organisational skills, ignorance of current evidence and guidelines, and a lack of feedback regarding one’s own clinical behaviour proved to be insurmountable. CA was still seen as the diagnostic gold standard by most interviewees.

**Conclusions:**

Oversupply and overutilisation can be assumed to be present in study regions but are not immediately perceived by clinicians. The problem is unlikely to be solved by regional collaborative initiatives; optimised resource planning within the health care system combined with appropriate economic incentives might best address these issues.

**Supplementary Information:**

The online version contains supplementary material available at 10.1186/s12913-024-10904-5.

## Background

In Germany, the rates of coronary angiograms (CAs) performed are considerably higher than in comparable countries [1, 2]. Within Germany, regional variation is large [3]. For example, based on health insurance data, 4–5 times as many CAs are performed in regions with the highest insurance coverage (17–18 per 1,000 insured people) compared with regions with the lowest insurance coverage (3.9–7.6 per 1,000 insured people) [4]. It has been suggested that many CAs in Germany lack clinical justification [2, 4]. It should be noted that patients with myocardial infarction or acute coronary syndrome (ACS, acute stage) are excluded from the analysis. In this case, CAs are indicated (‘effective care’) and a reduction in the number of CAs is not desirable. The current guidelines, however, restrict the use of CA for diagnosing stable coronary heart disease (CHD). On the other hand, non-invasive management of stable CHD has been shown to be underused in Germany [5]. Numerous studies have shown that in patients with chronic stable CHD, percutaneous intervention (PCI) can alleviate symptoms but does not improve prognosis regarding myocardial infarction (MI) or cardiovascular death [6, 7].

Chest pain is a common reason for consultation in primary care, with a prevalence of 0.7–2.7% [8, 9]. Self-limiting conditions as well as life-threatening disease can cause this symptom. Only 10–15% of patients presenting with chest pain in primary care have CHD as the underlying pathology [8, 10]. Most scenarios can be adequately assessed by considering patient history, physical examination and, where appropriate, non-invasive methods [11]. While these steps are covered by the current guidelines, treatment pathways are a potential means to improve implementation.

Treatment pathways are defined as multidisciplinary treatment plans that structure care for a defined clinical problem [12, 13]. They are meant to translate the current evidence into the regional health care systems. Their main objectives are to improve the quality of care, to ensure resources are utilised appropriately and to reduce variation in the care provided [14]. Ertner and Anwand [15] proposed an iterative 12-step model for pathway development, the ‘pathway clock’. Their framework covers the preparation, development and implementation of a regional pathway. We adapted this pathway clock to our context and used it for our process planning. We aimed to evaluate the development and implementation of regional treatment pathways to improve care of patients with suspected stable CHD, especially regarding the diagnostic use of CA.

## Methods

### Study design and context

The main objective of the KARDIO study^1^ was to investigate CA use for suspected stable CHD in Germany. Two sub-projects examined regional variation of CA use [3] and potential determinants. Here, we report on a before-and-after study to prove the concept of joint-developed regional treatment pathways for patients with suspected CHD. More specifically, we explored the barriers and facilitating factors in the development and implementation of treatment pathways in four high-utilisation regions.

### Treatment pathway development

#### Selection of regions and study participants

Based on data from three major German health insurance funds, we identified study regions with a high rate of CAs in non-acute situations. Specifically, we chose regions within the top quintile of CA utilisation in non-emergency cases as study sites (*n* = 80). The final selection of four study regions among high-utilisers was based on pragmatic aspects (research infrastructure available), as well as balancing sociodemographic characteristics such as urban versus rural regions in eastern and western Germany as well as in northern and southern Germany. In three out of the four chosen regions, the selected region was surrounded by other high-utilisation areas. In each region, general practitioners (GPs) and specialists in cardiology, radiology, nuclear medicine, and emergency medicine were invited to participate in developing a treatment pathway for patients with suspected stable CHD.

In each region, three treatment pathway development meetings with regional physicians were planned over a period of 6 weeks. The desired group size and composition was six GPs, three cardiologists, three hospital-based cardiologists, four emergency physicians and two radiologists/nuclear medicine specialists. Each session was estimated to last about 1.5 h. Members of participating academic departments in the respective region facilitated the meetings.

Before and after the closure of a treatment pathway development session, we asked the physicians in the respective regions whether they would be willing to evaluate the results. For this endeavour, we used the same mailing lists as for recruiting treatment pathway developers.

#### Theoretical framework and qualitative guide

Complex interventions are widely used in social and health care. In 2000, the Medical Research Council (MRC) published a framework for researchers to develop and evaluate complex interventions [16], which has since been updated [17, 18]. To guide the development process, we drew on the treatment pathway clock suggested by Ertner and Anwand [15]. It has been used to develop regional treatment pathways in Germany and includes the iterative components of the MRC framework.

#### Process planning of treatment pathway development

Meetings were scheduled as described in Table 1. If necessary, the participants were able to arrange additional meetings or working groups for specific tasks. To provide a framework for discussion, we established an algorithm proposed by the national guidelines [19]. However, we aimed for a participatory approach and therefore respected decisions made by the group, even if they conflicted with the current guideline recommendations.

**Table 1:** Planning process for treatment pathway development

1. Meeting	2. Meeting	3. Meeting
Explanation of the KARDIO study; introduction of participants; identification of referral pathways; exchange about current diagnostic/therapeutic approach or existing regional standards	Short review of the first meeting; drafting a treatment pathway by developing subcategories; presentation and provision of working materials (national guidelines on CHD and S3 guidelines on chest pain, including decision support material).	Merging the results; further discussion and finalisation of the treatment pathway and facultative patient materials; reflection on current and future standards and potential barriers.
=>> Development of a regional treatment pathway =>>		

### Data collection

We collected data from participant observations and interviews to elucidate the process of treatment pathway development and implementation.

#### Observation

All treatment pathway development meetings were observed by trained researchers who took notes but did not actively engage in the discussion [20]; pathway moderators took field notes after each meeting (participating observation). Written material on flip charts or boards was photographed [21, 22].

#### Interviews

The interviews were conducted 1 month after completion of the treatment pathway. We focused on the development of the treatment pathways, the attitudes and experiences of the participating doctors towards the diagnostic process and the obstacles and facilitating factors in the implementation of the treatment pathway. We approached physicians who had participated in the development of treatment pathways and who were also expected to apply them in their own practice (pathway developers). We also interviewed physicians who were not involved in the development of the treatment pathway but who took part as pathway users. We used a semi-structured interview guide to explore the participants’ expectations and attitudes towards the treatment pathway and its implementation. Data collection was conducted by telephone interview or on site at the participants’ offices. We audio recorded the interviews and transcribed them verbatim.

### Data analysis

The data analysis was conducted by nine individuals with various backgrounds (physicians, health scientists, and study assistants) at three study sites. The members took part in an initial training session covering the coding tree, which was based on the interview guide. The coding tree was modified iteratively in shared video meetings. All text material was coded at least twice in a consensual approach; disagreements were resolved in group discussions. The data were analysed by following the qualitative content analysis described by Kuckartz [22] and by employing the content analysis software MAXQDA [23].

## Results

In one of our four initial study regions, only a small number of physicians and hospitals could be recruited to participate in treatment pathway development. Therefore, we had to replace this region with another.

### Study regions

Table 2 provides geographic characteristics of the study regions investigated. All regions were high utilisers of CAs.

**Table 2:** Geographic characteristics of the four study regions

Region	Description	Urbanity	Geography
1	City in a metropolitan region	Metropolitan region	Western Germany
2	City with rural periphery	Mixed urban and rural	Southern Germany
3	City with rural periphery	Mixed urban and rural	East Germany
4	Rural district	Rural	Central Germany

### Physician participants

We conducted interviews (*n* = 45) with physicians who were involved in the development of the treatment pathways (*n* = 22) as well as physicians who agreed to use the treatment pathways after being introduced to the project (*n* = 23). While the majority of the participants worked in the ambulatory sector (*n* = 31), a smaller proportion (*n* = 14) worked in several hospitals (*n* = 7). Additional characteristics of the respondents are summarised in Table 3.^2^

**Table 3:** Characteristics of the interviewed physicians from the ambulatory sector*

Demographic data	
**Gender** n (% of available)*	
Male	20 (64.5%)
Female	11 (35.5%)
**Age in years** mean (range)*	
Male	52 (38–65)
Female	53 (41–64)
**Specialty** n (%)	
Primary Care	26 (83.87%)
Cardiology	5 (16.13%)
**Interview duration in minutes**	
Range	6.67––31.70

* Sociodemographic data not available for hospital-based participants

Although we contacted all eligible physicians and institutions in the four regions by email, and later by telephone, there was limited willingness to participate in the study. The low participation in the study affected the treatment pathway development as well as implementation. Lack of time or having too many commitments were given as reasons. During the course of the study, the COVID-19 pandemic began, making recruitment and conducting the study even more difficult.

### Treatment pathway development

#### Compliance and commitment

In the treatment pathway meetings, we did not reach the desired number of participating physicians and it was not possible to form balanced groups regarding disciplines and/or primary versus secondary and/or ambulatory versus hospital care. Irregular participation and lack of commitment from physicians made progress difficult. Table 4 shows the exact numbers of participating physicians.

**Table 4:** Date and number of treatment pathway sessions and number of participants per specialty

Region	Number of treatment pathway sessions	Cardiologists (n)	General practitioners (n)	Hospital-based cardiologists and emergency physicians (n)	Radiologists or nuclear medicine specialists (n)
1	I	8	7	1	2
	II	5	7	1	2
	III	7	5	1	1
2	I	1	4	3	1
	II	1	4	3	2
	III	0	5	2	2
3	I	0	3	1	3
	II	0	1	2	3
	III	1	3	2	0
4	I	0	4	1	0
	II	1	3	0	0
	III	0	1	0	0

Key actors in the care of patients with suspected CHD often could not be persuaded to continuously participate in the meetings. In some cases, this made physicians, who had previously taken part, cancel their participation in subsequent meetings. Indeed, nuclear medicine specialist 1 commented, ‘developing a treatment pathway without the cardiologist is nonsense’ (field notes), during a treatment pathway development meeting.

#### Rationale of the treatment pathway

The information that their region was chosen for being in the top quintile of CA utilisation in non-emergency cases (no ACS) provoked intense discussion. Not all the participants agreed with this and considered the frequency of CAs in their area to be appropriate. Some even suspected an undersupply. Others saw oversupply and overutilisation as a problem, but not in their own region.

*There are areas in Germany where everybody is being taken to the cathlab, irrespective of symptoms or findings. […] We’re not doing that at my current hospital.* (Hospital based cardiologist 1 [interview])

Physicians who perceived an overutilisation of CAs mentioned that economic incentives were the main reasons.

*[…] In the end it wasn’t about patient welfare, [but] rather hospital finances.* (GP 1 [interview])

*It’s well known that here in Western Europe we do the most catheter examinations. It may be well that in some cases it is actually exaggerated, because it is also clearly advantageous economically. […] there is a big economic role.* (Hospital-based cardiologist 2 [interview])

#### Characteristics of regional treatment pathways

In all regions, the working groups produced a document outlining recommended care for patients with suspected CHD, including the roles of primary and specialised care providers. Two regions developed an algorithm with concomitant comments, while two regions produced explicit recommendations. One region created a short version as a tool to give an overview of the treatment pathway in an abbreviated form. In no case was the idea of a computerised decision aid for CA taken up, which had been developed and made available to the working groups.

### Content of the developed treatment pathways

Choosing the guidelines on which the regional treatment pathway should be based was controversial in some regions. Some participants, especially cardiologists, considered the European Society of Cardiology (ESC) guidelines to be the more relevant than the national guidelines on CHD. For a detailed comparison of the algorithms created with the national and ESC guidelines, please see the supplementary material.

#### Relevance of stress ECG

Some participants disagreed with the national guidelines on when to use the stress ECG diagnostic procedure. The national guidelines assign a low diagnostic value to this test. Many physicians who contributed as developers or users favoured stress ECG as a routine examination, despite some being aware of the high false-positive rate (Cardiologist 1) of this test.

*Well, I think that in the case of symptoms, in symptomatic patients, a stress ECG is an important and correct examination. If the findings are clear, invasive testing would immediately follow.* (Cardiologist 2 [interview])

#### Pretest probability/Marburg heart score

Some GPs were unaware of symptom scores, nor were they familiar with the notion of pretest probabilities for suspected CHD and their consequences for further work-up. More common seems to be risk stratification based on known cardiovascular risk factors, such as smoking or hypertension. The interviewees expressed reluctance to use these scores in their own practice, despite recommendations by guidelines and regional treatment pathways. Time constraints were also mentioned as a reason. In addition, some GPs felt uncomfortable with a diagnosis (of inclusion or exclusion) based solely on history and physical examination. They assumed their patients expected technical investigations.

*Difficult to practically implement, […] are these scores for estimating CHD probability. […] That– that just takes too long, right? […] And the patient, who has symptoms of some kind, or a possible idea of what diagnosis should look like, is also not satisfied when I explain to him ‘But now you only have a very low CHD probability’, is he? He wants certainty, right?* (GP 2 [interview])

#### Shared decision-making (SDM)

The national guidelines for CHD recommend SDM for invasive testing, for which evidence-based patient materials and decision aids have been made available. However, these decision aids were not accepted let alone implemented in any region. SDM was not even mentioned in any of the developed treatment pathways. In two study regions, written patient information was included to promote and support the informed, active role of the patient. The physicians expressed discrepant views, with some saying they discuss management informally with patients and invite them to participate in the decision. Others emphasised that patients expect relevant decisions to be made by their physician. The following quotes document discrepant attitudes to patient participation.

*[…] Patient information is still the most helpful thing; in the end the patient makes the decision, and this is how it should be.* (GP 2 [interview])

*In this treatment pathway the patient’s wishes are just not sufficiently considered.* (Cardiologist 3 [interview])

*[…] And– yes. […] One aspect which I experience every day, and which was not definitively […] discussed, is that it is mostly up to the patients themselves whether they participate in the diagnostic work-up, right?* (GP 3 [interview])

*Apart from the fact that many patients don’t necessarily expect or want to participate, they traditionally, especially the older ones, they want me to decide, don’t they?* (GP 2 [interview])

#### Perceived value of CA as a diagnostic test

A CA was widely regarded as a ‘standard examination’ (Hospital-based cardiologist 1) or ‘the gold standard in diagnosis’ (GP 1 and Hospital-based cardiologist 2), with a procedural risk considered to be low compared to the perceived benefit.

*[…] And then a coronary angiography is done easily; it’s not very invasive. You just give contrast medium and prick the wrist once if everything goes as planned.* (Hospital-based cardiologist 3 [interview])

Sometimes, non-invasive testing seems to be omitted out of convenience.

*Patients with chronic CHD being put [on the catheterisation table] without demonstrated ischaemia, that happens every day. It [omitting non-invasive demonstration of ischaemia] doesn’t help anybody, does it? That’s why I think that far too many CAs are being performed.* (Cardiologist 1 [interview])

While both the national guideline CHD and ESC guidelines recommend non-invasive diagnosis of CHD and limit the role of CA to clarification of therapeutic options, the participants saw CA primarily as a diagnostic test.

*This is a relatively good procedure, it is […] easy to use, meanwhile it has very few side effects. I think it’s worse if you miss a case of CHD than if you do one coronary angiogram too many and the result says ‘Yes, everything’s good’.* (GP 4 [interview])

*[…] And sometimes there are also non-heart patients who need some kind of reassurance… so that the symptoms can simply calm down. […] I have treated patients who did not have CHD in the first place, and [performed CA] three or four times because I simply had to reassure [the patient] or because I had to check it again.* (Hospital-based cardiologist 4 [interview])

*[…] [CA] shows me the extent of CHD and I obtain guidance for future management. I only get that with coronary angiography.* (Hospital-based cardiologist 4 [interview])

The latter view was an exception: most participants did not differentiate between CA as a diagnostic test and the evaluation of management options as the current guidelines recommend. As an extreme case, a cardiologist used CA repeatedly, up to four times, to reassure the patient.

#### Attitudes towards guidelines

It became apparent that the participants had inconsistent attitudes towards guidelines in general. Some doubted that a standardisation of diagnostic methods could do justice to the individuality of patients. Some regarded guidelines as institutions restricting their own and their patients’ autonomy.

*I actually found my own way [in treatment].* (GP 4 [interview])

Others maintained that guidelines varied from their lived professional experience, and that instead they should follow their established routine. Still other physicians welcomed standardisation of management, especially for their younger or less experienced colleagues.

*You have to rely on your instincts when seeing a patient, to be honest.* (Cardiologist 4 [interview])

*[…] that’s definitely good for colleagues who have little experience or are at the beginning of their careers, that they definitely have a recommendation for action that they can rely upon. […]* (GP 5 [interview])

Most cardiologists from our sample believed they were already acting in strict accordance with the guidelines, thus making a new treatment pathway irrelevant.

*[…] For us, this treatment path has no real use because we strictly adhere to the guidelines of the German Society of Cardiology.*^3^ (Cardiologist 2 [interview])

Many GPs stated that they were not familiar with any guidelines relevant to suspected CHD. The interviewed physicians from all disciplines were not aware of a difference between their own behaviour and the treatment pathway recommendation. Consequently, they felt no need to change their current practice.

*So, we were already working according to this […] or a similar treatment pathway before. So not much has changed now, has it?* (Cardiologist 4 [interview])

*Well, as I said, we’re basically doing this, or a lot of this, already. We don’t have to change anything.* (GP 5 [interview])

## Discussion

In four regions with high utilisation of Cas, we supported the development of treatment pathways to improve the management of patients with suspected CHD. Against the background of likely overuse of CAs and underuse of non-invasive testing, we expected that treatment pathways developed based on established guidelines would improve diagnostic workup in primary and secondary care. Treatment pathway development received little support in all four regions, and it is unlikely that the treatment pathways developed could lead to improved practice. The participants expressed very little commitment to change. Although this attempt clearly failed in all study regions, our experience provides relevant insights into the process of evidence appraisal and implementation.

### Barriers against the clinical treatment pathway

We regard the following findings and mechanisms as relevant for the failure of the treatment pathways investigated in our study.

The participants did not have the resources to coordinate regional stakeholders’ work on a common project. In addition, they did not have the necessary management and organisation, skills and sufficient time. Although academic departments could provide these to a certain degree, they did not suffice within the available time frame. Moreover, working in close proximity to each other did not necessarily help. In one region, cardiology consultants from two hospitals in the same city had not met in person before. However, in some cases, the participants lacked the will and the necessary motivation to develop a treatment pathway. The participants did not view high regional utilisation of CAs as a problem. Therefore, there was limited motivation to improve care through a collaborative effort.

There were also knowledge gaps that made it difficult to reflect on one’s own practice regarding the work-up of patients with suspected CHD. The participants regarded CA as a test required to diagnose CHD. This stands in contrast to the current guidelines in which the diagnosis of CHD is based on non-invasive tests. The participants were reluctant to rely on patient history for decisions with potentially grave implications. This is understandable in a medical culture that values technical procedures more than patient history and clinical findings [24].

The participants expressed their reservations regarding guidelines and similar attempts to standardise clinical practice. Many of the interviewed GPs were unfamiliar with the actual guideline recommendations, while the cardiologists tended to selectively acknowledge them. Guidelines were not welcomed as a means to make scientific evidence accessible to clinicians; rather, they were viewed as an infringement upon clinical freedom. These findings should remind us that although clinical mindsets change, they do so at different speeds depending on geography and culture. On the other hand, the participants admitted to acting according to the current guidelines. A self-critical attitude towards one’s own clinical behaviour was not widespread in our sample of physicians.

Some of the participants mentioned financial interests. Given the association of CAs performed and the high number of catheterisation facilities at the national level, influence must be regarded as likely [3]. The number of catheterisation labs is not regulated in the German health care system. If capacity exceeds demand, which is suggested by the high number of Cas performed in Germany compared with other countries, then induced demand would be an important mechanism driving CA frequency.

Although funding constraints afforded only a brief timeline, we doubt that additional time available for treatment pathway development would have led to different results.

### Implications

A regional treatment pathway developed collaboratively between physicians is unlikely to solve the problem of over-supply and over-utilisation. Many physicians and hospitals would have to act against their own financial interests, which cannot be expected. Health economic incentives should be created to encourage the use of diagnostic alternatives to CAs within the system. Decisions to restrict catheterisation facilities must be made and enforced at the level of the health care system.

We regard these barriers against successful treatment pathway development and implementation as fundamental. Although the literature on effective guideline implementation is extensive [6, 25], the simple addition of more tactical components would not have led to success, nor would additional personnel or monetary resources.

Ideally, path development should be a bottom-up activity. With the active participation of regional stakeholders, a treatment pathway can be tailored to the available health services, thereby increasing its acceptance and implementation. Under these conditions, evidence and guidelines can be better implemented and adapted to practice [14]. The initiative reported here, however, was of a top-down nature. The regional stakeholders had limited motivation to learn and implement a new approach. In this and a parallel study [26], we found that regional professional standards were dominated by specialist groups not interested in a change of practice.

Recently, it has been decided that coronary computer tomography (CCTA) will be covered by German health insurance as an alternative to CA. CCTA could satisfy the expressed need to see the extent to which stenosing CHD is present. However, it would offer the possibility of treating patients which single-vessel disease conservatively with medication prescriped by a GP and referring patients with multi-vessel disease or main stem stenosis to specialists. We expect considerable changes regarding diagnostic assessments of suspected stable CHD.

### Strengths and limitations

A strength of the study is that we selected four different high-care regions based on nationwide routine data, considering the urban/rural aspect and geographic variation. The consistency of the results among the four regions can also be seen as a strength of this investigation. Although recruitment in the study was not satisfactory, our data analysis yielded similar findings in the four regions, suggesting that we achieved saturation. Although we cannot exclude that non-participants would have provided additional relevant insights, we had very rich data regarding criticism and sensitive themes.

The small number of participating physicians is a limitation. Given the confounding between study participation and willingness to critically reflect on one’s own practice, the difficulties we encountered with recruitment are a relevant finding. The COVID-19 pandemic, which added considerable stress to the physicians’ work environments, was partly responsible for the low willingness to participate. The desire to take part in a research project, both through collegial discussion and interviews, was clearly affected by these exceptional circumstances. Another limitation was a small amount of financial support and the short time frame, so a pilot study could not be performed. Nevertheless, the study setting allowed us to collect comprehensive qualitative data addressing opportunities and barriers regarding the care of patients with suspected CHD.

## Conclusions

Based on the high rates of CAs, we attempted to develop regional treatment pathways for patients with suspected CHD. We chose four regions with high utilisation rates. This attempt failed predominantly due to motivational and attitudinal reasons. The reactions by the participating physicians suggest that they are more than comfortable with the regional oversupply. Collaborative management pathways cannot solve the problem of induced demand because the participants would have to act against their own material interests. A lack of treatment capacity is immediately felt by everybody (e.g., waiting lists), but the effects of oversupply are insidious, such as diagnostic creep [27, 28]. Solutions must be developed at the level of the national health care system by adjusting existing economic incentive structures. In Germany, a first step has been taken with the introduction of a new billing code encouraging stress ECG as an alternative to or prior to performing CA.

### Electronic supplementary material

Below is the link to the electronic supplementary material.

Supplementary Material 1

## Data Availability

Because the data may provide enough information to allow participants to be identified, the cites have been anonymised.

## Footnotes

1 Linksherzkatheter bei Brustschmerzen und KHK: KARDIO-Studie (G-BA 01VSF16048).

2 N.B. in Germany, specialists are based both at a hospital and in the community.

3 These guidelines do not exist: the German Society of Cardiology endorses the ESC guidelines. European member societies and colleges are discouraged from developing their own guidelines.

## Abbreviations

ACS: Acute coronary syndrome
CA: Coronary angiograms
ECG: Echocardiogram
ESC: European society of cardiology
GP: General practitioner
MI: Myocardial infarct
MRC: Medical Research Council
OECD: Organisation for Economic Co-operation and Development
PCI: Percutaneous intervention
SDM: Shared decision-making
