# Supplementary material for: Development and implementation of a treatment pathway to reduce coronary angiograms - lessons from a failure
Source: BMC Health Serv Res. 2024 Apr 25;24:527. doi: 10.1186/s12913-024-10904-5 (PMC11046897; doi:10.1186/s12913-024-10904-5)
Supplement: Supplementary file 1 — Supplementary Material 1 [file 12913_2024_10904_MOESM1_ESM.docx]

Supplementary Files'

Figure S1

Framework for the development and evaluation of the treatment pathway according to the iterative pathway clock.(15).


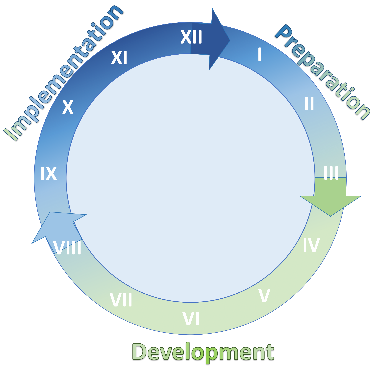


The pathway clock describes a development and evaluation circle, an iterative process regarding complex intervention. It consists of the phases preparation, development, and implementation. Of those, the development was conducted in group discussions with regional physicians (highlighted green), while preparation and evaluation were conducted by the study team (highlighted blue). In detail the phases consist of:

1. Preparation: Development and identification of an intervention in which the target disease (I: suspected coronary heart disease), the target group (II: patients in three settings with suspected coronary heart disease), and the scope (III: diagnostic and therapeutic approach) are determined.

2. Development in group discussion: Feasibility assessment of the intervention in which the entry (IV) into the actual pathway development takes place. During path development, the interfaces (V), typical treatment processes for patients (VI), and responsibilities (VII) are discussed and adapted (VIII).

3. Implementation: During the development meetings a concrete time table (XI), in which the implementation of the treatment pathway, was determined. Further evaluation was conducted by the study team regarding points deviation (IX) and implementation difficulties. The evaluation (XII) comprised of qualitative physician interviews regarding their experiences with the pathway development and implementation

Supplementary File

Table S1

*Table: Semi structured interview guide*

| **Theme: Development and consensus** |
| --- |
| How did you experience the treatment pathway development/meetings? |
| How or did the treatment pathway/outcome/agreement influence the collaboration? |
| What is the informal/implicit norm (subjective norm) in the region? |
| How diagnosis is specifically ascertained among patients with suspected CHD in your region? |
| What do you think of the treatment pathway/outcome/agreement in terms of content? |
| What is your attitude regarding the treatment pathway/outcome/agreement overall? |
| **Theme: Attitudes and experiences regarding diagnostic process** |
| Please explain which investigations for CHD are most important for you. |
| What is your opinion about the national guideline CHD? |
| **Theme: Treatment pathway implementation (inclusion barriers and facilitators)** |
| Has your medical practice changed as a result of the treatment pathway? What has changed? |
| Describe how you succeeded in implementing the treatment pathway/outcome/agreement in your daily routine. |
| Did you have the impression that you were able to implement the things you had planned in the treatment pathway/outcome/agreement? |

Supplementary File

Table S2

*Table: Treatment pathway content compared to guideline recommendations regarding diagnostic approach*

| **Content/Recommendation** | **National guideline CHD** | **ESC** | **R1** | **R2** | **R3** | **R4** |
| --- | --- | --- | --- | --- | --- | --- |
| **Diagnostic approach** |  |  |  |  |  |  |
| Definition of acute versus stable symptoms | No | No | No | No | Yes | Yes |
| Assessment of pre-test probability with established scores | Yes | Yes | Yes | Yes | Yes | Yes |
| Pre-test probability according to various risk factors for CHD | No | Yes | Yes | Yes | Yes | Yes |
| Is there a diagnostic stop when risk is below 5% or 15% respectively? | Yes (15%) | Yes (5%) | No | No | Yes (5%) | Yes (5%) |
| **Functional non-invasive testing** |  |  |  |  |  |  |
| Exercise electrocardiogram in every stable diagnostic situation | No | No | No | Yes | Yes | No |
| Stress electrocardiogram to rule out CHD in case pf Pre-test probability (ptb) between 15% and 30%. | Yes | No | No  But consider in ptb < 15% | - | - | Yes |
| Stress echocardiogram | No | No (+/-) | Yes | No | Yes | No |
| Dobutamine stress MRI | Yes(-) | No (+/-) | No | No | No | No |
| Myocardial perfusion scintigraphy | Yes (-) | Yes (-) | Yes | No | Yes | No |
| **Anatomical non-invasive testing** |  |  |  |  |  |  |
| CT calcification score in every stable diagnostic situation | No | No | No | No | No | No |
| CT calcification score in specific Pre-test probabilities between 5 to 50% | Yes | Yes | No | Yes | No | No |
| **Invasive testing: coronary angiograms** |  |  |  |  |  |  |
| Diagnostic Intervention  *Neither guideline recommends invasive testing diagnostic purposes* | No | No | Yes | Yes | Yes (-) | No (?)  no differentiated representation |

*Table: Treatment pathway content compared to guideline recommendation regarding patient involvement in decision making*

| **Content/Recommendation** | **National Guideline CHD** | **ESC** | **R1** | **R2** | **R3** | **R4** |
| --- | --- | --- | --- | --- | --- | --- |
| **Patient involvement** |  |  |  |  |  |  |
| Shared decision making explicitly mentioned | Yes | No | No, respectively: no differentiated representation | | | |
| Use of patient decision aids (DA) |  |  |  | | | |
| Use of patient DA in diagnostic situation | Yes | No | No differentiated representation | | | |
| Use of patient DA in therapeutic situation | Yes | No | Not applicable  No, respectively: no differentiated representation | | | |
